# Effects of chronic physical exercise on executive functions and episodic memory in clinical and healthy older adult populations: a systematic review and meta-analysis protocol

**DOI:** 10.1186/s13643-024-02517-0

**Published:** 2024-04-01

**Authors:** Soledad Ballesteros, Michel Audifren, Andreea Badache, Vera Belkin, Christoforos D. Giannaki, Antonia Kaltsatou, Uros Marusic, Mohammad Mosaferi Ziaaldini, Manca Peskar, José M. Reales, Jennifer A. Rieker, Pinelopi S. Stavrinou, Juan Tortosa-Martinez, Claudia Voelcker-Rehage, Yael Netz

**Affiliations:** 1 Department of Basic Psychology II, UNED, Madrid, Spain; 2 University of Poitiers, CNRS, Paris, France; 3 School of Health Sciences, Örebro University, Örebro, Sweden; 4 Institute of Sport and Exercise Sciences, University of Münster, Münster, Germany; 5 School of Life and Health Sciences, University of Nicosia, Nicosia, Cyprus; 6 Department of Physical Education and Sport Science, University of Thessaly, Volos, Greece; 7 Institute for Kinesiology Research, Science and Research Centre Koper, Koper, Slovenia; 8 Ferdowsi University of Mashhad, Mashhad, Iran; 9 Department of Methodology, UNED, Madrid, Spain; 10 Department of Psychology, Faculty of Life and Natural Sciences, University of Nebrija, Madrid, Spain; 11 Department of General and Specific Didactics, Universidad de Alicante, Alicante, Spain; 12 Department of Psychology and Ergonomics, Faculty V: Mechanical Engineering and Transport Systems, Technische Universität Berlin, Berlin, Germany; 13 The Levinsky-Wingate Academic Center, Tel-Aviv, Israel; 14 Department of Health Promotion and Rehabilitation, Lithuanian Sports University, Kaunas, Lithuania

**Keywords:** Diseased older adults, Episodic memory, Executive functions, Healthy older adults, Three-level meta-analysis, Exercise

## Abstract

**Background:**

Executive functions (EFs) and episodic memory are fundamental components of cognition that deteriorate with age and are crucial for independent living. While numerous reviews have explored the effect of exercise on these components in old age, these reviews screened and analyzed selected older adult populations, or specific exercise modes, thus providing only limited answers to the fundamental question on the effect of exercise on cognition in old age. This article describes the protocol for a systematic review and multilevel meta-analytic study aiming at evaluating the effectiveness of different types of chronic exercise in improving and/or maintaining EFs and long-term episodic memory in older adults.

**Methods and analysis:**

The study protocol was written in accordance with the Preferred Reporting Items for Systematic Reviews and Meta-Analyses (PRISMA) guidelines. Several databases will be searched. Randomized controlled trials (RCTs) conducted in older adults aged ≥ 60 years providing any kind of planned, structured, and repetitive exercise interventions, and EFs and/or episodic memory measures as outcomes, published in English in peer-reviewed journals and doctoral dissertations will be included. Two independent reviewers will screen the selected articles, while a third reviewer will resolve possible conflicts. The Cochrane risk-of-bias tool will be used to assess the quality of the studies. Finally, data will be extracted from the selected articles, and the formal method of combining individual data from the selected studies will be applied using a random effect multilevel meta-analysis. The data analysis will be conducted with the metafor package in R.

**Discussion and conclusion:**

This review will synthesize the existing evidence and pinpoint gaps existing in the literature on the effects of exercise on EFs and episodic memory in healthy and unhealthy older adults. Findings from this meta-analysis will help to design effective exercise interventions for older adults to improve and/or maintain EFs and episodic memory. Its results will be useful for many researchers and professionals working with older adults and their families.

**Systematic review registration:**

PROSPERO CRD42022367111.

## Introduction

### Background

Developed nations are experiencing unprecedented increases in the population of older adults mostly due to the reduced birth rates and the increased longevity of their citizens. The latest projections by the United Nations suggest that the global population could grow to around 8.5 billion in 2030, 9.7 billion in 2050, and 10.4 billion in 2100 [1]. More importantly, it was estimated that in the European Union, the old-age dependency will increase from 29.6% in 2016 to 51.2% in 2070 [2].

With respect to brain and cognition, aging is the main risk factor for neurodegeneration with prevalence increasing further with age [3]. Given the demographic situation and the relation of aging with cognitive decline, there is great interest in exploring effective ways to improve and/or maintain cognitive functions for independent living [4]. The main approaches to improving brain functionality and cognition in older adults are physical activity (PA), cognitive training, and social engagement [5]. The focus of this paper is PA.

Colcombe and Kramer [6] conducted two decades ago a seminal meta-analytic study on the effect of aerobic fitness on cognition in older adults. The study included 18 intervention studies and showed robust benefits in cognition with the largest fitness-induced benefits occurring for executive control processes, as previously hypothesized by Kramer, Hahn et al. [7]. The magnitude of the effect was moderated by the length of the training intervention, the length of the training sessions, the type of the intervention, aerobic training or aerobic combined with strength training with better results for combined training, and the gender of the participants with larger benefits for women.

The research conducted since then has provided compelling evidence that regular practice of PA can promote and/or maintain cognitive and brain functioning in late adulthood and old age [8–10]. The literature usually distinguishes between PA and exercise. The former entails any bodily movement produced by skeletal muscles that increases energy expenditure relative to rest. Exercise is a subcategory of PA that is planned, structured, and repetitive and is more specifically designed to improve one or more components of fitness: cardiorespiratory fitness, flexibility, balance, coordination, strength, and/or power [11].

The main objective of this review focuses on analyzing the effect of various exercise interventions, including aerobic exercise, strength straining, dance, and balance exercises on executive functions (EFs) and episodic memory of older adults. There is agreement among aging researchers that significant declines appear with age in EFs [8, 12] and long-term episodic memory, related to intentional retrieval of episodes [13, 14]; thus, several studies focus on these components [15–18]. EFs are formed by a series of effortful top-down cognitive processes necessary for mental and physical health, success in life, and cognitive, social, and psychological development [19]. The dorsolateral prefrontal cortex (DLPFC) plays a crucial role in the different components of EFs [20] and contributes to these components via functional connectivity with different brain regions [21].

Improvements in fitness are expected to improve EF processes such as coordination, inhibition, planning, and updating of working memory [7] but also cognitive flexibility as well as higher-order executive functions related to reasoning and fluid intelligence. Inhibitory control refers to the ability to control one’s attention and do what is more appropriate in each circumstance. Moreover, inhibitory control allows us to selectively attend to a certain stimulus suppressing other stimuli. Self-control is another aspect of inhibitory control related to resisting temptations and avoiding impulsivity. Inhibitory control declines greatly in normal aging [22], and older adults struggle to avoid distractions [23]. A recent cross-sectional study has showed that the EFs inhibition, shifting, updating, and dual tasking decline in healthy older adults but not with the same intensity with inhibition showing the greatest decline and dual tasking the smallest [24].

Working memory (WM), and more particularly updating of WM, is another key EF that serves to hold verbal or visual-spatial information in mind that is no longer perceptually present and working with it [25]. WM and inhibitory control are closely related and often support one another. The decline in WM with aging correlates with a decrease in the speed of information processing in older adults [26, 27].

The third component of EFs, cognitive flexibility, builds on working memory and inhibitory control. Flexibility means to being able to adjust to changed demands and to change perspectives, task switching, and set shifting. Cognitive flexibility is a property of the cognitive system that helps us to pursue complex tasks [28]. An additional component of EFs is higher-order EFs which is related to reasoning, problem-solving, and planning and is synonymous with fluid intelligence [19].

Episodic memory is a key cognitive process that allows us to represent past experiences and employ these representations to serve current and future goals [29, 30]. It is one of the earliest memory systems that decline with increasing aging. Impaired episodic memory with aging, involving retrieval of personal experiences and their spatial and temporal contexts, is well documented in the literature [31]. At the brain level, the medial temporal lobe and the hippocampus play a crucial role in retrieving information from episodic memory [32].

Since the influential meta-analytic study conducted by Colcombe and Kramer [6], the effect of exercise on EFs and episodic memory has been examined in numerus meta-analyses [33–47]. However, some reviews included only healthy populations [39, 40, 46], while others included only cognitively impaired or demented older adults [34, 35, 41, 43, 44, 48]. Chen et al. [36] included both healthy and cognitively impaired older adults but not demented. While one review examined only nursing home residents [38], another review [45] included only community-dwelling older adults. On the other site, while one review [33] focused only on aerobic exercise, another review [37] centered merely on resistance training, yet a third one [42] focused on exergames.

The current study addresses the gaps of the existing literature and aims to extend the knowledge of the effect of exercise on the principal components of cognition in old age. Our comprehensive review will potentially include healthy and non-healthy older adults and a wide range of exercise modes. This argument stems from a gap in evidence-based literature as pointed in a recent article [49]. For example, it has been argued that research on older populations is typically biased towards healthy and relatively young older adults, with certain groups of older individuals frequently being excluded from research on aging — especially in studies with physical activity interventions [49]. Such a review will pose a general question on the effect of exercise on cognition in advanced age (a general effect size will be calculated) followed by examining the moderating effect of various exercise modes (e.g., aerobics, strength, balance), several exercise characteristics (e.g., intensity, frequency, length), and a wide range of population characteristics (e.g., education level, percentage of females, health status), protocol characteristics (e.g., type of control group, type of analysis — intention-to-treat *vs.* per-protocol), and exercise settings (community dwelling and nursing homes). In addition, the present review will make an in-depth examination of the moderating effect of the outcomes used to assess cognitive functions, distinguishing, for example, working memory span indexes (e.g., number of correct responses in reading span tasks) from updating working memory indexes (e.g., error rate in n-back tasks), the latter requiring much more executive control than the former. The choice of adequate indexes of EFs is a very sensitive problem when estimating the effect size of the influence of regular exercise on EFs.

To summarize, the main objective of this systematic review and meta-analysis is to address the gaps encountered in the existing literature and to investigate the advantages of a broad range of exercise interventions on two key cognitive components, EFs and long-term episodic memory, across diverse groups of older adults and considering very selective outcomes. The findings from this review will be instrumental in developing effective training methods to enhance EFs and episodic memory in healthy and unhealthy older adults.

## Methods and analysis

The protocol of this review was prepared following the Preferred Reporting Items for Systematic Reviews and Meta-Analysis Protocols (PRISMA-P) 2015 statement and Cochrane systematic review methodology [50, 51]. The protocol is registered on the *International Prospective Register of Systematic Reviews* (number CRD42022367111).

**Fig. 1:**
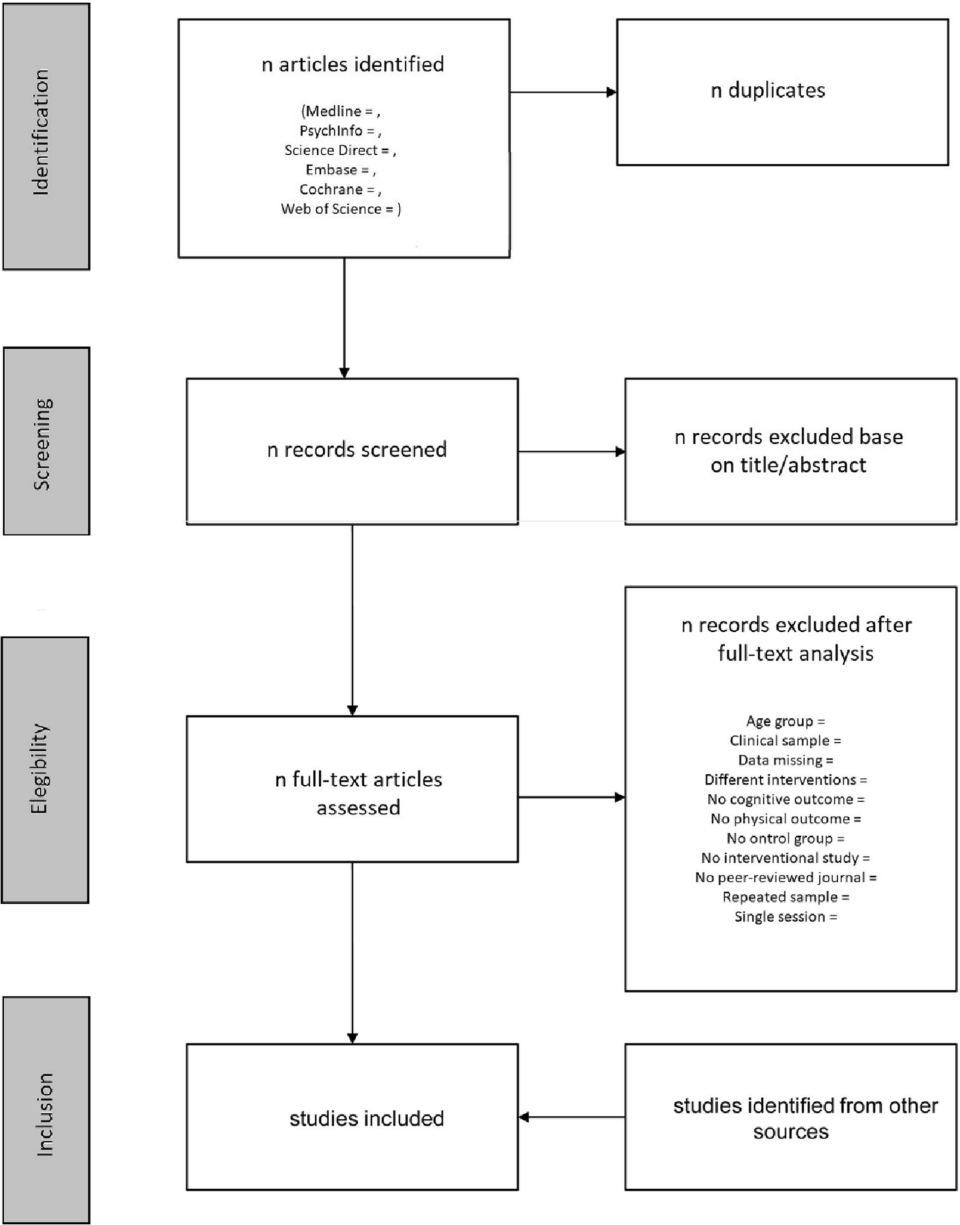
Flow chart diagram of the search strategy of the systematic review

Figure 1 presents the planned flow chart of the systematic review and meta-analysis with a summary of the selection process.

### Eligibility criteria

Eligibility criteria follows the PICO framework regarding population, intervention, comparator, outcome, and study type.

#### Population

The study will include participants with a mean age of 60 years or older and a minimum age of 50 years. It will include both healthy older adults and older adults diagnosed with various conditions such as mild cognitive impairment (MCI), Alzheimer’s disease (AD), or Parkinson disease (PD).

#### Intervention

Any randomized controlled trial (RCT) focusing on the effects of any type of exercise will be screened for inclusion, including aerobic, resistance training, coordination training, and other exercise programs such as tai chi, qigong, dancing, and exergaming. Several main exercise characteristics (type, session duration, session intensity, session frequency, intervention duration) will be assessed.

#### Comparators

Comparators will include light exercise, stretching, meditation, relaxation, and/or passive control groups (waiting list, treatment as usual, and habitual activities).

#### Outcomes

Cognitive outcomes will include objectively assessed cognitive domains of EFs (inhibition, working memory, cognitive flexibility, and high-level EFs) and episodic memory. These cognitive domains should have been assessed at baseline and at the end of the intervention through well-validated cognitive tasks and psychological tests. Regarding EFs, the outcomes for assessing inhibitory control may include the Stroop task, Eriksen-Flanker test, Simon task, global–local task, go-no go task, random number generation task, saccade-antisaccade task, and stop-signal task (STT). To assess working memory, the tasks and tests may include the Corsi block-tapping test, reading span task (RST), operation span task (OSpan), backward verbal digit span task, visuospatial N-back task, or tone monitoring task. To assess cognitive flexibility, the instruments may include the Trial Making Test (TMT), the Alternative Uses Task (AUT), Brixton Spatial Anticipation Test (BSAT), Delis-Kaplan Executive Function System (D-KEFS, subtests: the Trail Making Test, the Color-Word Test, the Tower Test, the design fluency test, The Sorting Test), Remote Associates Test (RAT), Implicit Relational Assessment Procedure (IRAP), attentional set-shifting task (AST), or Wisconsin Card Sorting Test (WCST). Among the instruments to assess high-level executive functions are the Raven’s Colored Progressive Matrices (RCPM) and Tower of Hanoi (TOH). In the case of episodic memory, the assessment tools may include the Rey Auditory Verbal Learning Test (RAVLT), SEMantic Episodic Memory Test (SEMEP), Wechsler Memory Scale (WMS; only the subtests that assess episodic memory), Hopkins Verbal Learning Test (HVLR-R), language-based paradigms, or the 360° video for episodic memory assessment. All the indexes of performance used for each of these tasks will be carefully selected to be sure that they reflect the targeted cognitive function (e.g., interference score in the case of the Stroop task, the Ericksen task, and the Simon task).

#### Inclusion and exclusion criteria

The inclusion criteria will be age (mean ≥ 60 with a minimum of 50 years), the practice of any type of physical exercise for at least 3 months, and provide outcomes including any EFs or episodic memory measure assessed at baseline (before physical training) and after training (post-training). If there were enough follow-up studies (e.g., 3 months, 6 months after training), they will be analyzed. Characteristics of exercise intervention such as frequency, intensity, type, and/or time of exercise (FITT) of the intervention program will have to be informed. Studies will be excluded if they do not meet the PICO conditions mentioned above, if they are not RCTs, if they do not have at least an active or passive control group, or if the written language is not English.

### Research questions

The present study is directed to answer six main research questions described below.

i. To what extent does exercise enhance EFs and episodic memory in old age (the global effect)?
ii. Do different types of chronic exercise (aerobic, resistance training, coordination training, and other exercise programs, such as Tai Chi or Qigong, dancing, or exergaming) have a different impact on EFs and episodic memory in older adults?
iii. Is the type of control group (active *vs.* passive) a moderator of the changes in the investigated cognitive domains?
iv. Is the effect of exercise different in healthy older adults and clinical older adults suffering neuropsychological disorders (MCI, PD, AD)?
v. Is age a moderator of the effect of exercise on the investigated components of cognition?
vi. Are duration and intensity of exercise moderators of the effect of regular exercise on cognitive aging?

### Literature search strategy

An initial search will be conducted at MEDLINE, Embase, PsychINFO, Google Scholar, EBSCO, SportDiscuss, CINAHL, Science Direct Dissertations, Web of Science, and Cochrane Central Registered of Controlled Trials (CENTRAL). These databases were selected because they are the most important and widely used to assure that relevant articles were not missed and in consultation with experienced researchers and librarians. Table 1 shows the detailed search strategy for PsychINFO. In addition, systematic reviews and meta-analyses published on episodic memory and the different EFs processes will be screened to check if the articles included in these publications should be considered in the present review.

**Table 1:** Search strategy for PsychINFO

Population	(elder* OR aging OR senior* OR (older adults) OR (older patients) OR (older women) OR (older people) OR (older persons) OR (older subjects) OR (old* age) OR geriatric* OR gerontolog* OR (late life) OR Alzheimer* OR Parkinson* OR (mean age of 60) OR (aged more than 60)
AND Intervention	( exercise OR (physical activity) OR (physical exertion) OR swim* OR gym* OR walk* OR danc* OR jog* OR run* OR cycl* OR bicycl* OR hiking OR (tai ji) OR (tai chi) OR yoga OR (qi gong) OR sport* OR (physical training) OR (strength training) OR (weight training) OR (resistance training) OR (balance training) OR (aerobic training) OR (anaerobic training) OR (endurance training) OR (muscle training) OR exergam* OR (active video game) OR Wii OR Kinect OR pilates OR feldenkrais OR (motor activity) OR (cardiac rehabilitation))
AND Outcome	(inhibition OR working memory OR executive function OR cognitive flexibility OR higher-level cognitive processes OR reasoning OR problem solving OR planning OR episodic memory OR Stroop test OR flanker task OR TMT task OR Wisconsin Card Sorting test OR flanker task OR global–local task OR go-no go task OR random number generation task OR saccade-antisaccade task OR Simon task OR stop-signal task)
AND Type of design	(random* OR RCT OR (clinical trial) OR (intervention* study) OR randomized control trial OR randomized trial)

Inclusion will be restricted to articles written in English published in peer-reviewed journals and doctoral theses. Studies published in other language will not be included. English is the most widely used scientific language to publish intervention studies and the language used in most systematic reviews and meta-analytic studies. Articles published from the inception will be considered for inclusion. An additional final search in the different databases will be conducted at the end of the review process to include more recently published studies.

After carefully reading all the retrieved articles, the data will be extracted for conducting the meta-analyses.

### Data extraction

Once the databases are searched, the retrieved articles will be exported in a Research Information Systems (RIS) format and imported into Rayyan [52], a web application created for article screening. The first step in Rayyan will consist of removing all the duplicates. Then, pairs of reviewers will work independently and blinded on screening articles based on title and abstract. Possible conflicts between the two independent reviewers will be solved by a third reviewer (J. M. R.). After completing the first selection stage by title and abstract, the next step will be retrieving the full articles corresponding to the included articles for careful reading. The idea is to extract in an Excel spreadsheet all the relevant information. The extracted data will include the following: (i) Characteristics: information regarding author(s), journal, publication year, and country; (ii) population: number of participants in each group, participants’ characteristics including mean age, sex, and clinical condition; (iii) interventions: including type of physical activity, intensity, session duration, total duration of the intervention, and adherence; and (iv) outcomes: in terms of tasks and psychological instruments used to assess memory and EFs, including sample size, means, and standard deviations at baseline and post-intervention and other possible time points corresponding (follow-up assessments) to the different (intervention and control) groups.

If a study will be relevant for our analysis but the data necessary to calculate the effect sizes will be missing or just the graphs were available, we will contact the corresponding author by email to ask for the relevant data. If the author does not respond, the missing data will be extracted from the graphs provided in the article using the online tool WebPlotDigitizer version 4.3.

In the case of RCTs with several time points, we will focus on the post-intervention at the end of the physical exercise training. If more time points or follow-up assessments were provided and enough articles contained assessments at 3 or 6 months after the end of the intervention program, the effects will also be considered. We will calculate Hedges’s *g* as the effect size.

### Risk of bias

The risk of bias (RoB) of each included study will be evaluated using the Cochrane ROB 2 tool [50, 53, 54]. Biases are assessed across five areas including randomization, deviations from intended interventions, missing outcome data, outcome measurement, and selection of the reported results. The risk of bias of each study will be assessed based on a series of questions provided for each of the five areas and the possible answers in the following five categories: “yes,” “probably yes,” “no,” “probably no,” and “no information.” Finally, the risk of bias in each area will be assessed as “low risk of bias,” “some concerns,” or “high risk of bias.” Teams of two reviewers will independently assess the risk of bias in the included studies. A third independent reviewer will resolve possible disagreements.

### Statistical analysis

Effect sizes (ES) will be modelled using a three-level structure because it is a better approach than a two-level structure when there are several dependent effect sizes in each independent study but only if the heterogeneity of the sampling variance is substantial. In three-level meta-analytic models, three different sources of variance are modelled. The third level represents the variance of effect sizes between studies; the second level describes the variance of effect sizes of the experiments, or measurements nested within each study; and the first level describes the sample variance. In the present study, we will perform a multilevel random-effects analysis using restricted maximum likelihood estimation. This analytical solution was designed to account for the nonindependence among effect sizes. This is the preferred methodology when the sampling variability is not too high. Heterogeneity among effect sizes (*I*^2^) will be assessed using the omnibus homogeneity test (Q), 0–40% indicates negligible heterogeneity, 30–60% indicates moderate heterogeneity, and 50–90% suggests substantial heterogeneity. A large *Q*-value means that differences between effect sizes do not derive from a common population mean from the study samples but are accounted for by other reasons.

The statistical analysis will be performed using rma.mv function of the metaphor package (version 2.4) [55] within the R software environment (version 4.0.1; R Core Team, 2021) [56]. The analytical steps provided by Assink and Wibbelink [57] will be followed. Dot-plot figures will be depicted using Mathematica (version 10.4) with software developed specifically for the present study.

To avoid outliers or influential cases that could distort the results of the meta-analysis, outlier and influential case diagnostics will be performed using the *influence* function of the metaphor package. The *influence* function calculates the influence of deleting one case at a time on the model fit or the fitted/residual values. Statistical heterogeneity will be assessed using the *I*^2^ test.

After a systematic publication search, it might occur that some studies were missed due to publication bias. That is, intervention studies that did not obtain significant results are not published, either because the authors did not submit them to a journal for publication or because the editor rejected them. We will address this important issue using two complementary statistics. The first explores the relationship between the precision and the observed effect size of the studies (the funnel plot and the statistical test of its asymmetry known as Egger’s regression test) under the assumption that effect sizes drive publication bias. In a funnel plot, the effect sizes are plotted against the standard error. An asymmetric funnel plot would suggest that publication bias exists, for example, an underrepresentation of nonsignificant results and/or negative effects on the bottom left side of the funnel plot. To evaluate the statistical significance of the funnel plots, we will apply the Egger’s test [58]. This test analyzes in a linear regression whether the standardized effect sizes can predict study precision, defined as the inverse of the standard error. The main goal of this analysis is to find a significant regression intercept that differs significantly from zero which would indicate a significant funnel plot asymmetry. We will also use the trim-and-fill method [59, 60] to determine the number of effect sizes that would need to be imputed to restore the symmetry of the funnel plot.

The second statistics we are going to use to assess publication bias is the P-curve technique, which assumes that publication bias is driven primarily through *p*-values, not by effect sizes. This relatively new methodology is based on the shape of the histogram of *p*-values, which depends on the sample sizes of studies and the actual effect size of the data. The method determines if the data estimates an actual, non-spurious effect size.

Once we had all the required information regarding the types of interventions, comparators, outcomes, and the healthy or clinical conditions of the participants of the finally included studies, we would be able to provide information regarding search results, descriptive results corresponding to studies and participants’ characteristics, overall effect size, and moderator analyses.

## Discussion and conclusion

The demographic data suggest that the world is aging very rapidly, and it is necessary to take actions against the cognitive decline that comes with aging. EFs and episodic memory are fundamental components of cognition that deteriorate with age and are vital for independent living. These cognitive declines significantly impact the performance of activities of daily living, independent living, and well-being among older adults. Previous reviews and meta-analyses screened and analyzed certain older adult populations [39, 46, 34, 41, 48], or specific type of exercise [33, 37, 42], providing limited answer to the question on the effect of exercise on EFs and episodic memory of older adults. The novelty of the present review is that it extends the knowledge about the effects of exercise on specific and central aspects of cognition to include different exercise modes and both healthy and unhealthy older adults.

Considering the key procedures and analyses, this systematic and meta-analytic review follows the PRISMA-P 2015 statement and the Cochrane systematic review methodology [50, 51]. The eligibility criteria of the articles to be included follows the PICO framework (population, intervention, comparator, and outcomes). Articles that met the inclusion criteria will be carefully read by pairs of reviewers who will extract the data for conducting the meta-analysis. Hedges’s g will be calculated as the effect size. Risk of bias of the included studies will be assessed with the Cochrane ROB 2 tool [50, 53, 54] by pairs of reviewers.

If the heterogeneity of the sampling variance is substantial, effect sizes (ES) will be modelled using a three-level structure. This approach is superior than a two-level structure. In a three-level structure, the third level corresponds to the variance of effect sizes between studies, while the second level refers to the effect sizes of the experiments within each study. Finally, the first level describes the sample variance.

The statistical analysis will be conducted using rma.mv function of the metaphor package (version 2.4) within the R software environment (version 4.0.1; R Core Team 2021), following the analytical steps of Assink and Wibbelink [57]. A specific software developed for the present study will be used to depict dot-plot figures. We will address possible publication bias using two complementary statistics, the funnel plot and the Egger’s regression test. The trim-and-fill method [59, 60] will reveal the number of effect sizes necessary to be imputed to restore the symmetry of the funnel plot.

The fact that this review includes only articles written in English may be a limitation. However, clearly, most studies are reported in English, and it is expected to extract very comprehensive information.

The central research question of this study is whether all training components recommended by official bodies are efficient for enhancing EFs and episodic memory and whether moderators, such as exercise program types and participants’ characteristics, could influence the effect size of the effect of regular exercise on cognitive aging [46, 61].

This systematic review and multilevel meta-analysis will provide evidence on how to optimize physical activity programs to improve and/or maintain these cognitive functions that decline more with age. So, the results of the present study would contribute to identify the gaps and limitations of current physical exercise research on executive functions and episodic memory in older adults. It would also allow to understand the quality of the research conducted to date in this field and summarize its main findings. The findings of this study will be useful for clinicians, physical trainer specialists, psychologists, social workers, and gerontologists, as well as older adults, their families, and wider public.

### Ethics and dissemination

This systematic review and meta-analytic study do not require approval from an ethics committee. The results will be disseminated in peer-reviewed journals and at international conferences and scientific meetings.

## Abbreviations

CIs: Confidence intervals
EFs: Executive functions
EM: Episodic memory
FITT: Frequency, intensity, type, and time of exercise
MD: Mean difference
PA: Physical activity
RoB: Risk of bias
SMD: Standardized mean difference
WM: Working memory
