# Correction: Effects of chronic physical exercise on executive functions and episodic memory in clinical and healthy older adult populations: a systematic review and meta‑analysis protocol

**DOI:** 10.1186/s13643-024-02545-w

**Published:** 2024-05-08

**Authors:** Soledad Ballesteros, Michel Audifren, Andreea Badache, Vera Belkin, Christoforos D. Giannaki, Antonia Kaltsatou, Uros Marusic, Mohammad Mosaferi Ziaaldini, Manca Peskar, Jose M. Reales, Jennifer A. Rieker, Pinelopi S. Stavrinou, Juan Tortosa‑Martinez, Claudia Voelcker‑Rehage, Yael Netz

**Affiliations:** 1 Department of Basic Psychology II, UNED, Madrid, Spain; 2 University of Poitiers, CNRS, Paris, France; 3 School of Health Sciences, Orebro University, Orebro, Sweden; 4 Institute of Sport and Exercise Sciences, University of Munster, Munster, Germany; 5 School of Life and Health Sciences, University of Nicosia, Nicosia, Cyprus; 6 Department of Physical Education and Sport Science, University of Thessaly, Volos, Greece; 7 Institute for Kinesiology Research, Science and Research Centre Koper, Koper, Slovenia; 8 Ferdowsi University of Mashhad, Mashhad, Iran; 9 Department of Methodology, UNED, Madrid, Spain; 10 Department of Psychology, Faculty of Life and Natural Sciences, University of Nebrija, Madrid, Spain; 11 Department of General and Specific Didactics, Universidad de Alicante, Alicante, Spain; 12 Department of Psychology and Ergonomics, Faculty V: Mechanical Engineering and Transport Systems, Technische Universitat Berlin, Berlin, Germany; 13 The Levinsky-Wingate Academic Center, Tel‑Aviv, Israel; 14 Department of Health Promotion and Rehabilitation, Lithuanian Sports University, Kaunas, Lithuania; 15 Department of Health Sciences, Alma Mater Europaea – ECM, Maribor, Slovenia

**Correction: Syst Rev 13, 98 (2024)**

10.1186/s13643-024-02517-0

Following publication of the original article [1], the authors reported that there was an error in the name of Manca Peskar and affiliations of Uros Marusic. Correct details were listed in the below table.

**Table:** 

Incorrect	Correct
Manca Pescar^7,12^	Manca Peskar^7,12^
Uros Marusic^7^	Uros Marusic^7,15^

The original article has been corrected.
